# Database of tensile test results of carbon fibers impregnated with thermoplastic polymer

**DOI:** 10.1038/s41597-026-07333-w

**Published:** 2026-05-02

**Authors:** Mikhail Lazarev, Denis Derkach, Fedor Ratnikov, Dilus Chukov, Sergey Gromov, Andrey Stepashkin

**Affiliations:** 1 HSE University, Pokrovsky Bulvar, 11, Moscow, 109028 Russia; 2 MISIS University, Leninskiy Prospekt 4, Moscow, 119049 Russia

**Keywords:** Composites, Mechanical properties

## Abstract

In this study, we present a comprehensive set of experimental data aimed at uncovering the mechanisms and regularities governing the deformation behavior of composites reinforced with continuous carbon fibers (CF) based on thermoplastic polymers. This work describes data extraction techniques that can later be used to optimize the mechanical properties of such structures using neural network models. This paper examines the thermoplastic polymer polysulfone (PSU) of the Ultrason S 2010 brand, which was used as the matrix material for the composites, while high-strength Toray T700SC fibers were used as the reinforcing fibers. Composite samples in the form of rods with a diameter of 1 mm were obtained by impregnating the fibers with a solution of polysulfone in N-methyl-2-pyrrolidone, followed by solvent removal. The collected dataset contains more than 600 tensile test results, including load-strain diagrams for different test conditions, data on the failure mechanisms of the specimens, and SEM images of the specimen microstructure in cross and longitudinal sections. This dataset will be useful for ML model development.

## Background & Summary

Composite materials are widely used in the development of highly efficient load-bearing structures due to their combination of low weight and high strength. The progress achieved over the last decade in areas such as aviation and renewable wind energy has been driven by the use of polymer composites reinforced with carbon, glass, aramid, and other types of high-strength fibers. This has made it possible to significantly reduce the weight of critical structures, allowing for a significant reduction in CO_2_ emissions and operating costs^1,2^.

Various thermosetting and thermoplastic polymers can be used as matrices. Thermosetting binders, such as epoxy or polyester, are currently the most widely used. When thermosetting matrices are used in the manufacturing process, polymerization occurs due to the interaction of the dosed components and temperature, ensuring the formation of the final product and the development of the properties of the material.

The advantages of thermosetting binders include low viscosity at room temperature, which allows effective impregnation of fibrous preforms without damaging them and ensures full utilization of the strength characteristics of the reinforcing fibers. Polymerization is generally not completed at room temperature, and upon subsequent heating, the binder becomes fluid again, which allows the final geometry of the composite product to be formed using thermocompression methods while removing excess polymer. Autoclave molding has proven to be the most effective method, providing minimum monolayer thickness and minimum porosity^3–5^.

The disadvantages of composite materials with thermosetting matrices include low shear strength, low impact toughness, and low crack resistance due to the limited deformability of the binders used, as well as high moisture absorption, which, under conditions of constantly changing temperatures and repeated crossing of the water freezing point, activates the initiation and further development of microcracks^6^.

Thermoplastic polymers can change from a solid state to a highly elastic and viscous state when heated, which makes it possible to recycle them and reshape products. Composites with matrices based on thermoplastic polymers are of considerable interest because, due to the high plasticity of the matrix, they can exhibit properties that composites with thermosetting matrices lack, such as high impact toughness, high crack resistance, and increased shear strength. In the case of high-performance polymers such as polyphenylene sulfide (PPS), polysulfone (PSU), polyethersulfone (PES), and polyetheretherketone (PEEK), they can also provide higher operating temperatures and increased thermal and chemical resistance^7–9^.

The technologies used to manufacture products based on thermoplastic polymers have a number of features, such as the high viscosity of their melts, which requires temperatures of 250–370 °C and pressures of up to 30 bar to achieve high-quality impregnation of fibrous preforms. Such processing conditions may distort the fibrous preform, and the reinforcing fibers may be partially damaged. At processing temperatures above 250 °C, partial degradation of the polymer material may also occur as a result of oxidation. All of this leads to a lower degree of realization of the reinforcing fiber properties and reduces the strength characteristics of the resulting composite^1,2,10^.

Polymers such as PPS, PSU, PES, and PEEK, which have high thermal and chemical resistance, are currently widely used for the production of composites. A significant number of studies are focused on the use of PEEK as a matrix for composite materials because of the excellent properties of this polymer at room temperature, as well as its high chemical and oxidation resistance^11–14^.

However, along with its advantages, PEEK also has a number of disadvantages, including its high cost and relatively low glass transition temperature of 145–155 °C. The transition to a highly elastic state at the glass transition temperature leads to a significant change in the properties of PEEK, while its mechanical and thermal properties can change by 3–10 times. For example, the elastic modulus can decrease by more than five times, and linear expansion increases significantly^14,15^. Therefore, the actual operating temperatures of composite materials based on PEEK are limited not by the onset temperature of its degradation, but by its glass transition temperature.

Other thermoplastic polymers can be used as a replacement for PEEK, offering lower cost while maintaining a high level of heat resistance and mechanical properties. One of the polymers we find attractive is polysulfone (PSU), which exhibits high heat resistance (up to 200 °C), good mechanical properties (tensile strength up to 80 MPa), dimensional stability, and a significantly lower cost, which may be useful for the production of widely used CF-reinforced composites.

Composites based on carbon fiber-reinforced polysulfone show stable elastic modulus behavior up to 160–180 °C^16,17^. In addition, the completely amorphous structure of PSU avoids the need for strict cooling rate control during composite production. Meanwhile, for PEEK-based composites, the crystallization kinetics of the polymer matrix can be an important factor affecting operational properties^18,19^.

The deformation behavior of materials with a thermoplastic matrix differs significantly from that of thermosets.

Elementary carbon and glass filaments are characterized by failure strains of less than 1%, and in threads consisting of such filaments, the elongation at failure usually does not exceed 2.5% and is comparable to the deformation of thermosets used as a matrix.

**Fig. 1:**
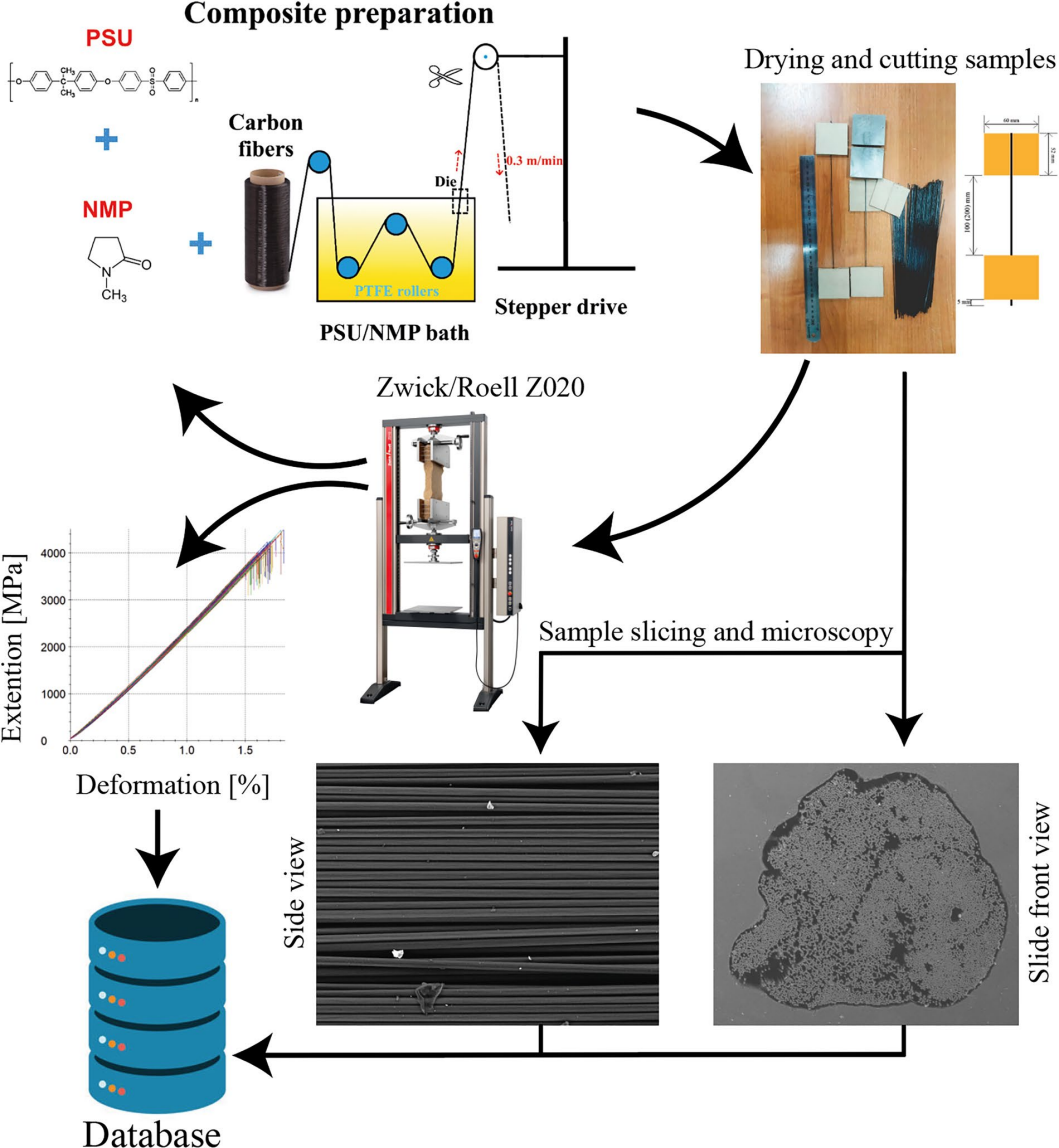
Pipeline of experiment and data collection.

In the case of a thermoplastic matrix, the strain at failure can reach tens of percent, which, even at relatively low stresses, can cause plastic flow in locally overstressed areas and eventually lead to local deformation and damage. Information on the deformation behavior of composite materials with thermoplastic matrices is necessary both for improving the structure of such composites and the methods used to produce them, and for refining the results of calculations for critical structures made of composite materials. This makes it relevant to create databases based on the results of experimental studies of the deformation and failure processes of such materials. The structure of our study is presented in Fig. 1.

With the advancement of machine learning algorithms and hardware capabilities, ML and deep learning (DL) methods have emerged as useful tools in materials science research. With available data sources, DL methods facilitate a new data-driven approach to research and application^20^. With current computational power, it is not a significant challenge to generate a dataset using computational methods based on physical models. In^21^, a deep learning hybrid convolutional neural network (CNN) and multilayer perceptron (MLP) model was developed to predict the mechanical properties of carbon fibers. The model was trained on synthetic data and successfully combined heterogeneous data inputs to improve prediction accuracy. However, simulations often do not match experimental data, which serves as the final validation of the scientific model.

Experimental data is critical for this validation. In^22^, the mechanical properties of a carbon-epoxy twill woven composite were characterized through tensile, shear, and compressive tests, providing valuable insights into real-world behavior, such as tensile and compressive moduli, strengths, and failure modes. However, the dataset does not contain morphological features of the studied materials, which are crucial for building predictive models. Another common problem is that even physical simulations may not match the experimental data, which serves as the final validation of the scientific model. Usually, in materials science, the quality and quantity of available data are the main obstacles to the use of DL methods, especially in real-world applications. Composite materials are no exception^23^. To the best of our knowledge, the data currently available on the internet is insufficient to build a reliable DL model for predicting the properties of a single composite wire^22,24^. In^25^, a data-driven approach was used to predict the mechanical properties of carbon fiber-reinforced composites by applying machine learning models to extensive empirical datasets.

In this study, we prepared a comprehensive dataset of tensile test results for carbon fiber-reinforced thermoplastic matrix composites (CFRP), comprising data from more than 600 experimental specimens. The dataset includes load-strain diagrams, fracture behavior, microstructural data on wire morphology, and details of the manufacturing process. The presented dataset can be used for machine learning (ML) applications aimed at predicting and optimizing the mechanical properties of such composites. By including different polymer-to-fiber ratios, the dataset provides a broad basis for training ML models to identify the underlying patterns and relationships governing material behavior.

The presented database and data mining algorithm demonstrate the feasibility of automating the extraction of meaningful features from tensile test results, making the dataset ready for further analysis. This work paves the way for future research on the use of ML methods to improve materials design, enhance composite performance, and reduce the trial-and-error phase of materials development. We believe that the dataset will serve as a starting point for researchers interested in applying ML models in composite materials science and related fields.

## Methods

**Fig. 2:**
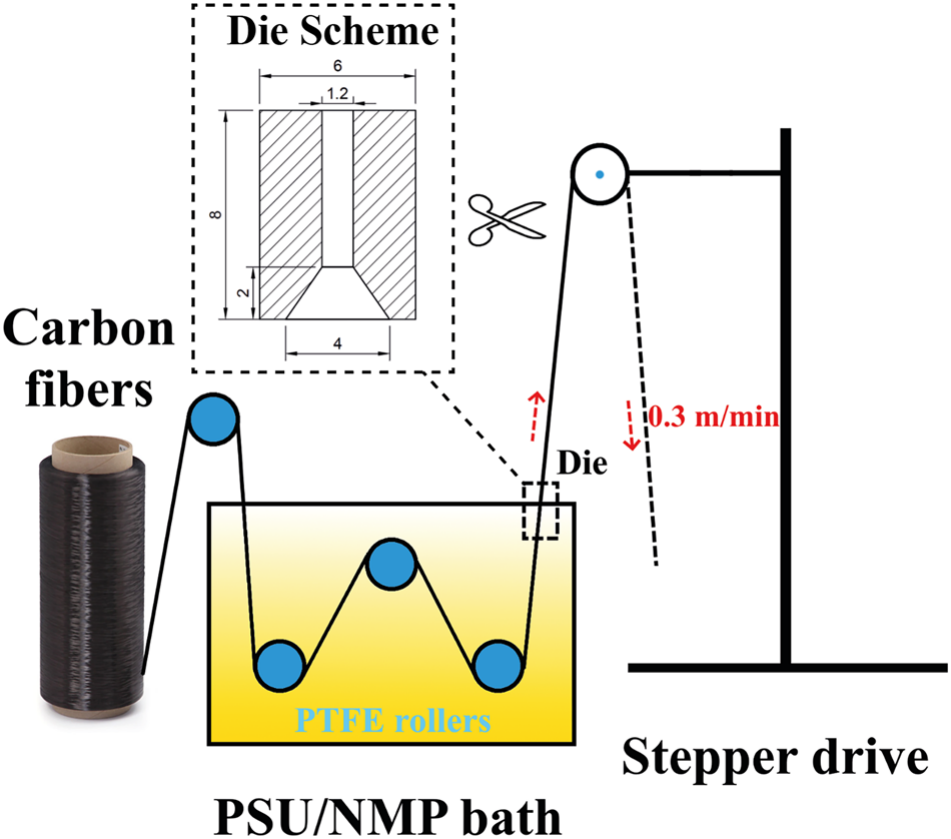
Scheme for obtaining polysulfone/carbon fiber microplastic samples.

The dataset was constructed based on the results of tensile tests of unidirectional carbon fiber-thermoplastic polysulfone composite samples. The composite samples were produced using a specially developed method, the scheme of which is presented in Fig. 2. The samples were obtained by impregnating carbon threads containing 12,000 filaments with a thermoplastic polymer solution, followed by solvent removal through drying.

High-strength carbon fibers Toray T700SC 12K (Toray Industries, Inc., Tokyo, Japan) were used to produce the composites. These fibers have an average filament diameter of 7.0 *μ*m, a density of 1.80 *g*/*cm*^3^, and a linear density of 800 tex. Their tensile strength is 4.9 GPa, and their Young’s modulus is 230 GPa. Polysulfone (PSU) Ultrason S 2010 (BASF, Ludwigshafen, Germany) was used as the matrix material. This polymer is amorphous, with a glass transition temperature (*T*_*g*_) of 187 °C, a density of 1.24 *g*/*cm*^3^, a tensile strength of 75.0 MPa, and an elastic modulus of 2.60 GPa.

The study used impregnation solutions of polysulfone Ultrason S 2010 in N-methylpyrrolidone (CAS: 872-50-4, molar mass: 99.13 g/mol, empirical formula C5H9NO), containing 20, 30, and 40 wt.% polymer. The solutions were prepared using an overhead stirrer (IKA EUROSTAR 40 digital) at a temperature of 50 °C; the mixing time required to homogenize the solution volume was 12 hours.

During the impregnation process, the carbon threads were pulled through a fluoroplastic bath filled with a solution of polysulfone in N-methylpyrrolidone, and the excess polymer was removed using a fluoroplastic die with a diameter of 1.2 mm. The linear speed of thread passage through the bath was 0.3 m/min. The resulting impregnated threads were cut into fragments 2 m in length and fixed vertically under a load of 0.5 kg. Primary drying was carried out in air for 24 hours, and final drying was performed at a temperature of 115 °C for 4 hours in a Binder FD-115 drying cabinet to remove part of the solvent.

**Fig. 3:**
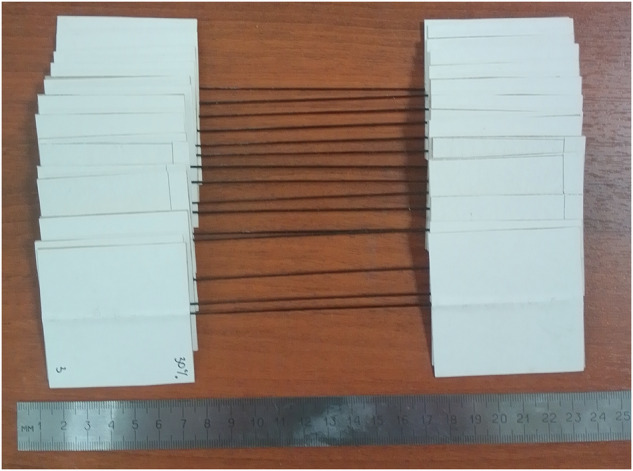
Samples prepared with 20% of a PSU solution.

The obtained samples had a diameter of 1.1 mm, and the polymer content varied from 18 to 40 wt.% depending on its content in the impregnating solution. For tensile testing, the impregnated samples were cut into fragments 230 mm long. Tensile tests were carried out on samples with a gauge length of 100 mm, with the ends glued into protective pads made of 1 mm thick cardboard measuring 52 × 60 mm. The samples were fixed in the cardboard pads using an epoxy binder. Samples prepared for testing are shown in Fig. 3.

For each sample, before gluing it into the cardboard frames, the polymer content was determined using an AND GR 202 analytical balance (AND, Japan). The polymer content in the sample was determined based on the difference in sample mass, while the fiber mass was calculated using the geometric and linear density data for a fiber of the given length.

The strength and deformation characteristics of the carbon fibers impregnated with a thermoplastic polymer were determined using a Zwick/Roell Z020 universal tensile testing machine (Zwick/Roell Group, Ulm, Germany) with a maximum applied force of 20 kN, equipped with a MultiXtens high-precision contact deformation measurement system. The tests were carried out in accordance with ASTM D4018 and ISO 10618. The total number of tested samples was 663.

Composite yarns were cut to length from the produced threads using a sharp blade on a flat surface to avoid fraying. Short segments were then prepared for cross-sectional observation and mechanically ground and polished to obtain a flat, mirror-like surface suitable for imaging. The prepared sections were subsequently examined by scanning electron microscopy (SEM) to document bundle packing, voids, and fracture-related features. Representative micrographs are provided together with the tensile data in the repository.

Porosity (void content) is known to strongly affect the mechanical response and failure mechanisms of composite materials. Direct porosity measurement for each tensile specimen was not feasible in our workflow, because gas pycnometry requires cutting a short segment (typically 20–30 mm), whereas the tensile specimens were prepared as 230 mm-long yarn fragments. Therefore, porosity was evaluated on representative short segments taken from the produced yarn batches using gas pycnometry. The measured porosity of the manufactured impregnated yarns was in the range of 12.5–18.5%. Since each yarn cross-section contains on the order of ~ 12,000 carbon filaments, the local void distribution within a cross-section is expected to be largely averaged out; thus, a batch-level mean porosity (together with the observed range) can be treated as a representative characteristic for the tensile specimens from the same batch. Nevertheless, because the measurement is destructive and performed on separate segments, porosity is not available as an individual per-specimen field in the released dataset. SEM and X-ray CT were used in this work for structural inspection and qualitative cross-checking, rather than for quantitative per-specimen porosity determination.

## Data Records

Our dataset^26^ consists of three files, and each of the file names starts with the respective concentration of a PSU solution: 20, 30, 40 mass.% The first sheet of the file contains summary information of the key properties of all samples. The columns in this sheet are as follows:

- Specimen Index - a unique identifier for each sample
- Young’s Modulus (in GPa) - the tensile was obtained from the stress-strain diagram
- Tensile Strength (in MPa) - the tensile strength was obtained from the stress-strain diagram
- Fiber concentration (wt. %) - the percentage of fiber content in the composite material
- Specimen length (in mm)
- Specimen diameter (in mm)
- Specimen mass (in mg)
- Pre-tension (1 N or 20 N) - when samples passed through the solution bath, a pre-tension was applied to prevent twisting during impregnation
- Die size (1.2 mm or 1.5 mm) - two different sizes of the die were used to regulate the removal of excess polymer
- Load (7.5 N and 20 N) - the solvent removal process was carried out by drying the impregnated threads in a vertical position under load
- Failure classification code - codes explanations provided in the section below.

**Fig. 4:**
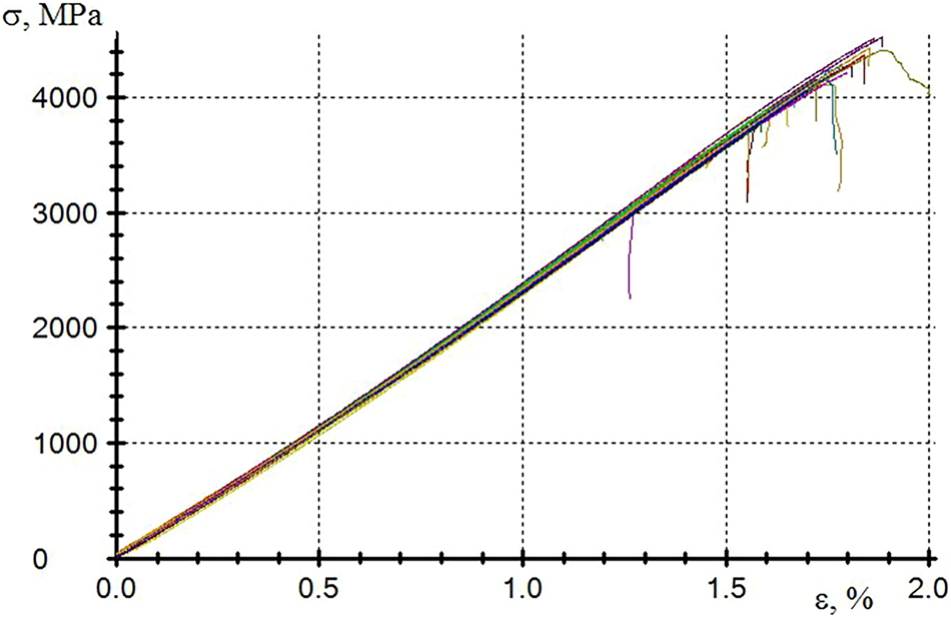
Typical form of the load-deformation diagram.

The remaining sheets correspond to each individual sample. Each sheet contains two columns: one for strain (%) and the other for stress (MPa). Additionally, each sheet includes a stress-strain diagram, providing a visual representation of the material’s behavior under tensile loading, an example of such a diagram is provided in Fig. 4.

During the tensile tests, a classification of the failure mechanisms of the obtained composites was performed. As a result, each sample was marked with a three-component failure type code, where the first symbol defines the type of failure, the second symbol the failure area, and the third symbol the failure location, respectively. This classification will allow for a more detailed analysis of damage mechanisms and improve composite manufacturing technologies. The first component of the failure classification code, representing the type of failure, can indicate several specific mechanisms: failure within the grip area, where the fracture occurs near the contact zone between the grips and the specimen, transverse fiber fracture, which occurs across the fiber orientation, longitudinal delamination, i.e. a separation along the fiber direction, tensile break (a complete break under tension), or other less common failure types. The second component, indicating the failure area, specifies the region where failure took place in relation to the grips. This can include within the grips, if the fracture is confined to the region held by the grips, along the boundary of the grips, i.e. where the specimen exits the grip area, at a distance less than the grip width away from the grips, across the specimen width, combined areas (spanning multiple regions), or other non-standard zones. The third component of the code, representing the failure location, provides the exact site of fracture along the specimen’s length. This can be at the top, bottom, or middle of the sample, a combined location, or an unknown location if the specific site of failure is indeterminate. This classification is summarized in Table 1. Examples of several destruction types are depicted in 8.

**Table 1:** Three-component destruction type cipher.

Type of destruction	Cipher	Failure area	Cipher	Failure location	Cipher
Grips	**G**	Inside the Capture	**I**	Bottom	**B**
Across the grain	**L**	Along the border of the grips	**A**	Top	**T**
Longitudinal delamination	**S**	At a distance from the grip not exceeding the grip width	**W**	Middle	**M**
Break	**X**	Width	**G**	Combined	**V**
Other	**O**	Combined	**V**	Unknown	**U**
		Unknown	**U**		

### Note on porosity

Porosity is not reported as a per-specimen field in the dataset because its measurement is destructive; instead, representative gas-pycnometry measurements on short segments indicate a typical porosity range of 12.5–18.5% for the produced yarns (see Methods).

## Data Overview

The database consists of tensile test results for composite threads, so the main variable is tensile strength. Its dependence on the manufacturing process is not immediately apparent. A typical load-deformation diagram consists of three characteristic sections that can be distinguished in the curves. In our case, these are the initial linear section up to 500 MPa, within which elastic deformation of the sample occurs, and the elastic modulus is 180–200 GPa; the final elastic section in the stress range from 2000 MPa to failure; and the transitional section, within which the elastic modulus changes due to the alignment of individual strands inside the thread. The main production parameters are the concentration of the polymer solution used to create the polymer matrix of the composite and the percentage of fiber content in the composite material.

**Fig. 5:**
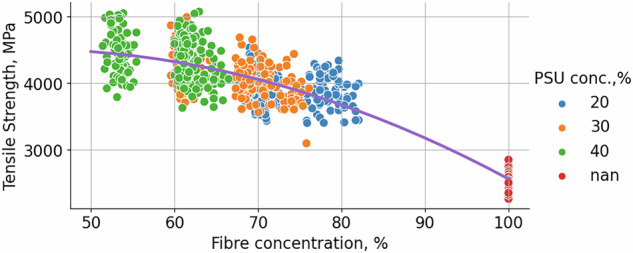
Tensile strength dependence on PSU and fiber concentration. The purple line is a quadratic fit of tensile strength.

As shown in Fig. 5, the tensile strength depends on the polymer content. At concentrations of 20 and 30 wt.%, the composites demonstrate the lowest tensile strength values, around 3900 MPa, which is due to insufficient stress redistribution in the material when local damage occurs under increasing load. Composites containing 40 and 50 wt.% polysulfone demonstrated the best mechanical properties, reaching 80–90% of the declared fiber strength in the composite. Increasing the polysulfone concentration above 50 wt.% may lead to a slight decrease in the average strength value, which is due to the appearance of thin layers of pure polymer in the structure of the material, affecting the degree of filler orientation and stress redistribution during testing. With an increase in solution concentration from 20 to 40 wt.%, an increase in the variability of the polymer-to-fiber mass ratio and strength values from sample to sample is observed. This behavior is associated with the high viscosity of the 40 wt.% impregnation solution, making it difficult for the polymer to penetrate into the internal volume of the composite due to its low fluidity, leading to reduced structural and property homogeneity.

**Fig. 6:**
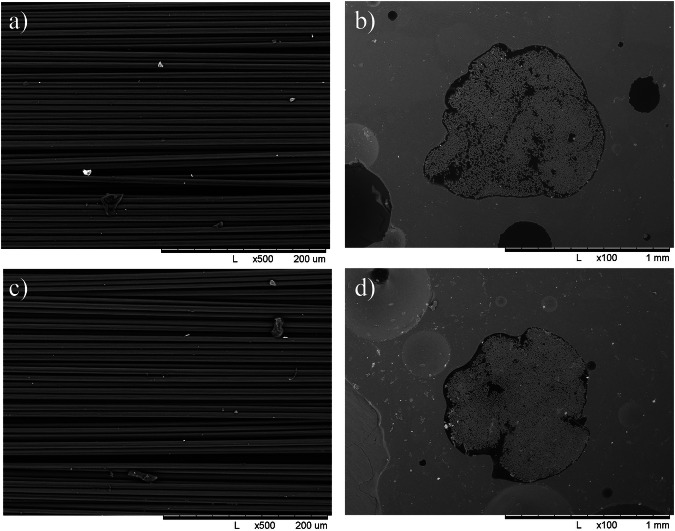
Microscopy images of two samples from side view (**a,c**) and cross section (**b,d**) with filament concentration of 64% and 72% (**c,d**).

Furthermore, to investigate the aforementioned dependence in greater depth, we conducted experiments with pure carbon fiber that was not impregnated with polymer. As a result, we found that the average tensile strength of these fibers was 2500 MPa. Additionally, we observed that the obtained relationship is well described by a polynomial. We fitted a second-degree polynomial using the fiber concentration % as a feature; the resulting curve is shown in purple in Fig. 5. However, for predicting the tensile strength of the manufactured composite, it is insufficient to use only this feature, as a significant scatter of values around the curve is observed. Therefore, it is necessary to examine the dependence of tensile strength on other parameters of the fiber production process. Figure 6 shows microscopy images of two samples. High-resolution images enable the identification of voids, delaminations, and fiber breakage patterns, which are essential for understanding the material’s mechanical response under tensile loading. Another interesting observation is that the figure shows two clusters of samples for each concentration of PSU solution. This is due to differences in the die size used to remove excess polymer. Experimental results show that, for samples with the same PSU solution concentration, a smaller die removes more polymer, thereby increasing the fiber content % in the composite.

**Fig. 7:**
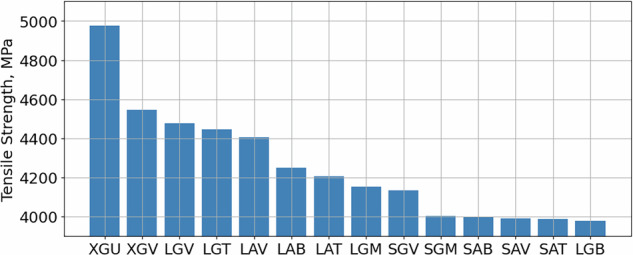
Mean tensile strength of samples for each destruction type cipher.

**Fig. 8:**
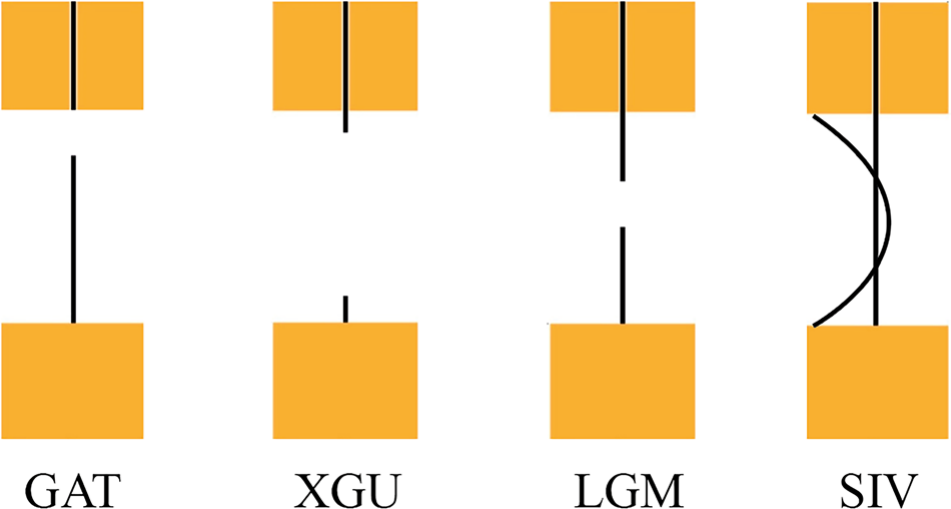
Three-component destruction type cipher.

Figure 7 presents a bar chart comparing the ultimate tensile strength of various composite samples, each categorized by a unique three-component failure code (Fig. 8). This coding system provides a detailed understanding of how each composite type responds under tensile stress, allowing for correlations between mechanical properties and specific failure mechanisms. From the chart, it is evident that the sample marked as XGU exhibits the highest tensile strength, reaching nearly 5000 MPa. Other samples, such as XGV and LGV, also demonstrate relatively high tensile strengths above 4400 MPa. In contrast, samples like SAV, SAT, and LGB show much lower tensile strengths, below 4000 MPa, indicating a different or weaker failure response. This distribution of tensile strengths among samples with varied failure codes highlights the variability in mechanical performance due to differences in failure mechanisms, which can guide further refinement in composite design and manufacturing processes.

## Technical Validation

### Standards, equipment, and conditioning

Tensile tests were performed on a Zwick/Roell Z020 with a MultiXtens extensometer in accordance with ASTM D4018 and ISO 10618. Specimen preparation and handling were standardised across batches (Section Methods), supporting measurement reproducibility over 663 tests.

### Sanity checks on curve shape and modulus

A typical load-deformation curve shows the expected three-region morphology with an initial linear-elastic part up to 500 MPa and an apparent elastic modulus 180–200 GPa, followed by a transitional region and final failure. The consistency of this shape and modulus range across specimens indicates stable alignment, gripping and gauge control.

### Label completeness and failure coding

Each specimen is assigned a three-component failure code at test time using a consistent procedure; codes distinguish valid tensile failures from events such as failure in grips and enable downstream filtering without deleting records. We verified the presence of codes for all records and provided aggregate counts per code (see Table 2). The coding legend is documented in Data Records.

**Table 2:** Number of specimen for each code in the dataset.

Type	Area	Loc	No.
S	G	V	331
S	A	T	73
L	A	T	64
L	A	B	47
S	A	B	47
S	A	V	30
X	G	V	29
L	G	V	13
L	G	M	6
S	G	M	6
L	A	V	4
X	G	U	2
L	G	B	1
L	G	T	1

### Cross-checks with imaging

Representative cross-section and side-view micrographs (see Fig. 6) document bundle packing and voids. To assess the spatial stability of the cross-sectional morphology along the yarn, we examined multiple longitudinal positions and found the cross-section appearance to be essentially constant along the length. This observation was corroborated by X-ray computed tomography of representative specimens, which did not reveal systematic longitudinal variations in cross-sectional geometry. Imaging preparation and SEM settings are described in Methods.

**Fig. 9:**
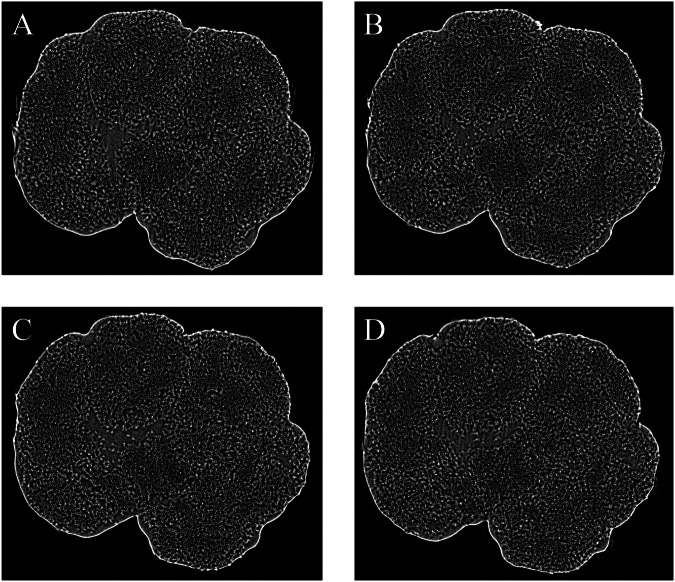
Reconstructed tomographic images of the sample: transverse sections (the section plane is perpendicular to the longitudinal axis of the sample). Sections (**A**–**D**) are selected along the sample axis with equal spacing: (**A**) in the first quarter of the length, (**B**) in the second, (**C**) in the third, (**D**) in the fourth (from one end to the other).

To assess whether the carbon filaments change their position along the sample height, we performed a tomography experiment to visualize their spatial distribution. The reconstructed cross-sectional slices are shown in Fig. 9. Comparison of the slices indicates that, in the bulk of the sample, the carbon filaments largely preserve their vertical alignment, indicating no appreciable rearrangement along the height. Small deviations from the vertical occur primarily in regions with a lower local filament concentration.

### Derived-value consistency

Derived fields (elastic modulus, ultimate tensile strength, strain at break) are computed from the raw curves using uniform definitions across all specimens. We checked units and basic value ranges for internal consistency.

### Scope of the dataset (temperature and orientation)

All tensile tests in this dataset were performed under ambient laboratory conditions and correspond to uniaxial loading along the yarn axis. The measured strain-to-failure is low (on the order of  ~1–1.5%), and the overall response is dominated by carbon filament fracture rather than thermoplastic matrix plasticity. Consequently, temperature-dependent and off-axis effects that are typically governed by the matrix (including ductile-brittle transition phenomena in thermoplastic composites) are not addressed by the present dataset and should be investigated separately if required for a given application.

## Usage Notes

While the dataset presented in this study represents a substantial step forward in the systematic collection of experimental data for carbon fiber-reinforced composites, it is important to recognize several limitations inherent in the current work. Although the explored material systems focus on a representative selection of high-performance thermoplastic matrices and carbon fibers, they do not encompass the full diversity of composite materials used in advanced structural applications. This restricts the generalizability of models trained on this dataset to other fiber-matrix combinations and environmental conditions, such as varying temperatures, cyclic loading, or exposure to corrosive environments. In particular, the dataset should not be used to infer temperature-dependent or off-axis behavior of thermoplastic composites, since all measurements were performed under ambient conditions in axial tension along the yarn direction. Expanding the dataset to include a broader range of conditions will be essential for the development of more universally applicable predictive models.

Moreover, the current approach relies predominantly on features extracted from tensile test results. While these features have proven valuable for initial machine learning efforts, they do not fully exploit the rich visual data available from images captured before testing, such as cross-sectional and side-view images of the composite samples. These images reveal critical information about the internal microstructure, fiber distribution, and surface characteristics that is not reflected in mechanical test data alone.

The dataset is most appropriate for within-system modeling, benchmarking of data-driven methods, and analysis of structure-property relations for solution-impregnated carbon fiber/PSU yarns. It should not be considered a universal training set for all carbon fiber/thermoplastic composite manufacturing routes.

## Data Availability

The current version of the dataset is available at 10.5281/zenodo.15502428. The dataset contains experimental results of the tensile test of carbon fibers impregnated with the thermoplastic polymer discussed in this paper; photos of the sample slices are also presented.

## Code availability

No custom code was used.
